# A qualitative study on the breastfeeding experiences of first-time mothers in Vientiane, Lao PDR

**DOI:** 10.1186/1471-2393-13-223

**Published:** 2013-12-05

**Authors:** Hope Mei Hong Lee, Jo Durham, Jenny Booth, Vanphanom Sychareun

**Affiliations:** 1 Melbourne University, the Nossal Institute for Global Health, Melbourne, Australia; 2 University of Queensland, School of Population Health, Centre for International and Tropical Health, Australia, Herston, Brisbane, Australia; 3 University of Health Sciences, Faculty of Postgraduate Studies, Vientiane, Laos

## Abstract

**Background:**

The benefits of breastfeeding are well-recognised. The majority of first-time mothers in the Lao People's Democratic Republic however do not follow WHO guidelines of exclusively breastfeeding for the first six months, and less than half breastfeed for two years. UNICEF identified lack of exclusive breastfeeding as the second highest risk factor for under 5 mortality in Lao PDR, closely following lack of skilled delivery care. This study explored the reasons and influences behind first-time mothers' breastfeeding practices, as well as the role of attitudes, beliefs and experiences in influencing those practices.

**Methods:**

A qualitative research design was chosen for this exploratory study. Two districts in Vientiane were selected, and in each district four focus group discussions, two with six first-time mothers and two with health staff were undertaken. In addition, sixteen in-depth interviews with first-time mothers and seven individual key informants were conducted.

**Results:**

Participants demonstrated positive attitudes towards breastfeeding and recognised its importance. Despite this, breastfeeding practices were suboptimal. Few exclusively breastfed for the first six months of the baby’s life and most of the first-time mothers included in the sample had stopped or planned to stop breastfeeding by the time the infant was 18 months of age. Work was named as one of the main reasons for less than ideal breastfeeding practices. Traditional beliefs and advice from health staff and the first-time mothers' own mothers, were important influences on breastfeeding practices. First-time mothers also cited experiencing tension when there were differences in advice they received from different people.

**Conclusion:**

Overall, the mothers were well-informed on the benefits of breastfeeding, and displayed positive attitudes towards it. Nevertheless, few maintained optimal breastfeeding practices in the first two years of the infant’s life. Further effort needs to be directed at addressing knowledge and non-knowledge barriers to optimal breastfeeding practices. Of particular importance is working with employers, developing supportive employment policies, providing postnatal support and working with lay people and health professionals. Research is also needed to identify the optimal combination of interventions to promote good breastfeeding practices.

## Background

Epidemiological evidence supports breastfeeding (BF) as an effective intervention to advance mother–child health. Particularly important is the timely initiation of BF (within 1 hour after birth), exclusive breastfeeding (EBF) for the first 6 months, and continued BF until the child is at least 24 months old (1-6-24 model) [1-4]. Breastfeeding benefits both the mother and her baby. For the baby, breast milk is highly nutritious, providing an excellent source of energy, protein, iron and vitamin A as well as antibodies and a variety of bioactive components to provide protection against disease [5]. Children who are breastfed have a decreased risk of life-threatening illnesses such as diarrhoea, ear and respiratory infections [6] and decreased risk for chronic diseases in later life [7]. If initiated soon after birth, BF helps the mother’s uterus to contract encouraging expulsion of the placenta and reducing the risk of severe bleeding and infection [8]. Other benefits include decreased risk of subsequent breast and ovarian cancers and hip fractures [9].

Despite the well-documented importance of EBF during the first six months of life, globally it is only a relatively small proportion of infants who are fed with EBF during their first 6 months [5,10]. In countries with the highest maternal and child mortality rates, prevalence of initiation of BF within one hour is only 48% and the prevalence of optimal BF practices in line with WHO guidelines is low [11,12]. In the Lao People's Democratic Republic (PDR), a lower–middle income country in South East Asia with high maternal and child mortality, the Ministry of Health has promoted BF since the mid-nineties [13]. Nevertheless, in 2004 low prevalence of EBF remained the second highest risk factor for under 5 mortality in the country, closely following lack of skilled delivery care [6]. A 2009 survey found only 26% of children under 6 months of age were EBF and only 48% were still BF at 20–23 months. Following the results of this survey, in mid-2009, the Ministry of Health, supported by UNICEF, launched a high profile Exclusive Breastfeeding (EBF) campaign [14].

While there have been some studies into postpartum practices in Lao PDR, the factors associated with BF practices remain under researched and in-depth, descriptive research around BF in Lao PDR is sparse. The objectives of the present study were to identify the reasons and influences behind the BF decisions of first-time mothers in Lao PDR. The study particularly focussed on decisions around when to initiate BF, timing of the introduction of complementary foods and BF duration.

## Methods

An exploratory, qualitative research design was chosen in order to obtain rich, in-depth information needed to understand and interpret the influences behind the target group’s BF decisions. The methods used were focus group discussions (FGDs) and individual in-depth interviews.

### Study setting

The study setting was two purposively selected districts in Vientiane Capital City. Vientiane Capital City consists of nine districts, with 700,000 inhabitants. Most of the villages (68%) are classified as urban (as defined by national statistical offices and calculated using World Bank population estimates and urban ratios from the United Nations World Urbanization Prospects [15]). Livelihoods are diverse and include a mix of people working in the public and private sectors including small-scale agriculture. Most of the population have relatively easy access to drug stores and 71% live within 10 km of a district health facility or community health centre [16].

The intent was to select districts within the City identified with low rates of optimal EBF practices. Only provincial level data were available however so the two included districts were purposively selected in discussion with the local authorities. The first district, Sisattanak is an urban environment, with a population of 68,195 while the second district of Hatxayfong is peri-urban (defined as an area in the process of urbanisation with a mix of rural and urban characteristics) and has a population of 78,385 inhabitants.

### Sample

**Figure 1:**
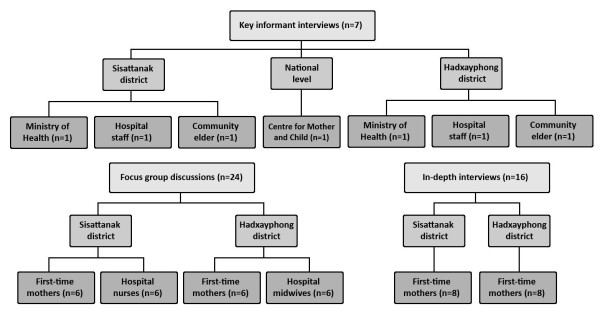
Diagram of sampling strategy.

The sample consisted of purposively identified first-time mothers, health staff including midwives, elders and key informants with participants selected for their ability to contribute to the research question. In total, four focus group discussions, two with six first-time mothers (one in Sisattanak district and one in Hatxayfong district) and two with health staff (one in Sisattanak district with nurses and one in Hatxayfong district with midwives) and sixteen in-depth interviews with first-time mothers were conducted (eight in Sisattanak district and eight in Hatxayfong district). A further seven individual key informant interviews were undertaken. These key informants included representatives of the Ministry of Health, health staff and respected female elders. In addition, one interview was conducted at the national level with a member of the Centre of Mother and Child Health (MCH), identified through a written request to the Centre. Figure 1 summarises the sampling strategy.

For the individual in-depth interviews and the two FGDs with first- time mothers, participants were recruited through referral from the relevant district health staff. Inclusion criteria for first-time mothers for the individual in-depth interviews and FGDs were: (1) aged 18 and over (2) first time mothers (3) delivered either at home or in a health facility; (4) first living child between the age of 7–18 months; (5) breastfeeding and (6) able to provide informed consent. First-time mothers who had not initiated BF were excluded as they would not have been able to address the research questions.

Women who had delivered at home were identified from district lists maintained by a network of health staff in each village. The age range for the mother’s first baby of 7–18 months was selected to allow the EBF period to be fully evaluated. The upper limit was chosen to avoid excluding too many potential participants, while minimizing the potential for recall bias. To reduce selection bias, the sample included participants with different levels of education, different employment and different socioeconomic status.

### Data collection

The in-depth interviews, key informant interviews and FGDs were all undertaken in Lao language with a translator and used a semi-structured interview guide. The intent was to gain an understanding of the reasons for decisions about EBF and BF practices. A semi-structured interview guide was with questions selected based on a review of the literature was used. Questions related primarily to initiation of BF, duration of BF and the introduction of complementary foods. Sociocultural influences and mothers’ personal experiences were also explored. The open response format of the questions in the guide allowed for probing to clarify responses and did not preclude respondents introducing other topics. The interviews and FGDs were facilitated by the lead researcher (HL) and a male doctor trained in qualitative methods from the University of Health Sciences, Vientiane. Field-notes and memos recorded interview dynamics and reflections on interview dynamics.

All of the women who agreed to participate in the in-depth interviews were interviewed in private to ensure confidentiality. For individual interviews, this occurred at a place most convenient for the individual. For the first-time mothers this was typically in their home. The FGD for the mothers in Sisattanak district was held in a private meeting room at the district hospital, and the FGD for the mothers in Hadxayphong was held in a private area at the local shrine. Key informant interviews and FGDs with health staff were conducted in private meeting rooms in the respective district hospitals. All interviews were transcribed in Lao based on the audio recordings and then translated into English. The transcripts were de-identified to ensure anonymity.

### Data analysis

The principal investigator (HL), with the principal supervisor (VS), coded the data reviewing the translated transcripts in English reading and re-reading and coding the data. Coded data was grouped into the key themes guided by the literature and included in the semi-structured question guide [17]. While the qualitative analysis used a pre-decided framework based on key themes identified in the literature, this did not exclude the possibility of new themes emerging [17,18]. Throughout the analytic process, there was a moving back and forward between the entire data set and the coded extracts.

## Ethics

Ethics approvals were obtained both from the Human Research Ethics Committee of the Melbourne University, Australia and the Ethics Committee of the University of Health Sciences, Lao PDR. In addition, prior to undertaking the study, the study design and purpose were discussed with the director of each district level hospital and their approval gained. Participants and the researchers did not know each other, but participants were aware of the interviewers’ status as researchers. Informed consent was gained from each participant after explanation of the study objectives, assurance of the confidentiality of their identity and assurance that choosing not to participate would not disadvantage them in any way. Consent was obtained from each participant to audio-record the interview.

## Results

The results of this study are presented in the following section under the themes identified in the literature and through our analysis.

### Demographics

The in-depth interview participants were between 21 and 39 years of age, with their first child between 7 and 18 months of age. Three of the mothers had competed primary level, five lower level secondary, five upper secondary and three tertiary. All were from the Lao Loum ethnic group. Table 1 summarises the age of the first-time mothers included in the in-depth interviews, the age of their baby at the time of the survey, the mother’s level of education and employment, place of delivery and district.

**Table 1:** Socio-demographic characteristic of the in-depth interview participants

**Respondent ID**	**Age of mother (years)**	**Age of child (months)**	**Education**	**Occupation**	**Delivery place**	**District**
IDI-1	27	18	Upper secondary	Unknown	Hospital	Sisattanak
IDI-2	26	10	Tertiary	Housewife	Hospital	Sisattanak
IDI-3	25	12	Tertiary	Housewife	Hospital overseas (Japan)	Sisattanak
IDI-4	39	7	Upper secondary	Housewife	Hospital	Sisattanak
IDI-5	26	10	Upper secondary	Factory worker	Hospital	Sisattanak
IDI-6	26	10	Tertiary	Shop worker	Hospital	Sisattanak
IDI-7	21	7	Lower secondary	Factory worker	Hospital	Sisattanak
IDI-8	21	13	Upper secondary	Housewife	Hospital	Sisattanak
IDI-9	20	16	Lower secondary	Unemployed	Hospital	Hadxayphong
IDI-10	22	7	Lower secondary	Housewife	Hospital	Hadxayphong
IDI-11	19	7	Lower secondary	Construction worker	Home	Hadxayphong
IDI-12	25	9	Primary	Farmer	Home	Hadxayphong
IDI-13	20	10	Lower secondary	Unemployed	Home	Hadxayphong
IDI-14	30	15	Primary	Unemployed	Hospital	Hadxayphong
IDI-15	23	8	Primary	Merchant	Hospital	Hadxayphong
IDI-16	23	10	Upper secondary	Housewife	Hospital	Hadxayphong

Thirteen mothers in our study delivered in hospitals. Those who gave birth at home did so with the assistance of a skilled birth attendant (SBA). All sixteen mothers attended antenatal care (ANC). Most of the mothers started attending ANC either in the first trimester or at the beginning of the second trimester and mostly on a monthly basis. Towards the end of their pregnancies, most were attending ANC weekly. All mothers, with the exceptions of the mother who delivered in Japan and the mother who had a caesarean section, followed the cultural tradition of lying on a ‘hot bed’ of embers postpartum, typically for 10–35 days. The ‘hot bed’ is a traditional practice in the immediate postpartum period in which hot embers are placed under the bed and herbs are added as an herbal remedy. In addition, most mothers drank traditional unsweetened herb tea during this period to stimulate milk production and followed a restricted diet influenced by cultural practices, with fruit and vegetables rarely eaten. Most of the mothers believed that following these traditional practices was important to ensure sufficient breast milk. Some of the midwives however felt that these dietary restrictions on fruit and vegetables could lead to insufficient breast milk. Other dietary restrictions were based on medical advice and included avoidance of fermented food, such as fermented pork meat with vegetable or fermented fish sauce. When asked how they felt about their breastfeeding experience overall the mothers in our study were typically very positive about BF and felt that they had “done the best for [their babies]” (IDI-4) by BF.

### Initiation of breastfeeding

#### Early initiation

Most of the first-time mothers were aware of the importance of early initiation of BF and the value of colostrum. Typically, they said that it was the health staff who assisted them in initiating BF usually within an hour of giving birth. According to one first-time mother however, it was very confusing in the delivery room: “the nurses had no time to teach [me how to BF]” (IDI-16) and her mother helped her initiate BF. For another of the first-time mothers there was a minor delay in initiation as a consequence of her having had a caesarean section. For another woman, although she planned to BF early, her breast milk did not come until early morning on the third day. In the meantime she “touched water to the baby’s lips” (IDI-6). Two mothers from Hatxayfong district reported a delay in initiating BF of over one hour, with one not starting BF until she returned home from the hospital about nine hours later. This mother reported that this delay was “normal” and a friend had done the same.

### Reasons for initiation BF

The main reason given by the first-time mothers for early initiation of BF was to make sure that the infant received colostrum. This was seen as having important immunological and nutritional properties. Most of the mothers also reported that the midwives or SBAs had encouraged them to BF. Several of the first-time mothers felt that BF strengthened the bond between the mother and child. As one participant explained “when a mother carries her child to breastfeed, they are close together all the time and can communicate in a special way”. Another first-time mother claimed an advantage of BF was than it saved money which would otherwise have been spent on milk formula.

In addition to health professionals and mother’s own beliefs, family elders (usually the mother’s own mother or mother-in-law) were identified as important influences on mothers’ EBF and BF practices. As one participant explained “my mother was the first one who motivated me to breastfeed, she talked about breastfeeding before the doctor did” (IDI-14). Rarely was advice reported as being given by the first-time mothers’ fathers. One elder also explained the importance of including both doctors and female elders (typically first-time mothers’ mothers) in decisions related to BF in order to avoid conflict between advice given by the elders and the advice given by health staff.

“If only the doctor said [do this] but the elder in the house did not agree, it will affect the mother…but if the doctor did not advise [to BF] and only the mother in the house, there [will] be conflict. Both the doctor and the elder in the house must cooperate” (KI-elderS).

This conflict became more apparent when exploring participants' experiences of EBF. This was because typically, the elders wanted to discard colostrum, delay initiation of BF or introduce others food before six months.

### Reasons for exclusive breastfeeding

Only five of the 16 first-time mothers included in the in-depth interviews (two from Sisattanak district and three from Hatxayfong district), reported EBF for the first six months (IDI-1, 7, 10, 12, 16). According to the two mothers from Sisattanak district, family and workplace support had helped them maintain EBF (IDI-1, IDI-7). According to one of these mothers, although her mother had suggested she should give water to her infant, she decided to follow her doctor’s advice to EBF instead (IDI-16). The mothers from Hatxayfong district did not mention specific family support in maintaining EBF but worked either in the home or on the family’s nearby agricultural plots. The 11 first-time mothers, who did not EBF for six months, were aware of the recommendation to EBF for the first six months. The main reasons given were difficulties with BF or based on advice from family, friends, elders or health staff they had decided to feed their babies complementary foods. Some also reported finding it difficult to manage both work and EBF. As one woman succinctly put it:

“We learn about theory, what is good and bad, but in real practice we cannot do the theory for giving breastfeeding- sometimes the mother has problems with money or illness- she cannot give enough milk to the baby- must give formula milk, must break EBF rules.” (IDI-4)

### Duration of breastfeeding

There was a wide range in the reported duration or planned duration of BF extending from one month up to two years, or “when the baby stops by itself”. The advice which first-time mothers received from health staff and female elders on optimal duration of BF was also inconsistent. Some reported being told that it “depends on the baby”, one was told 18 months, and others said they had been given no advice at all. As one person put it, “Generally Lao people say lots of different things about when to stop- 1 year 8 months, 1 year 2 months, 2 years… all different.” (IDI-3). Of the five mothers who reported EBF for the first six months, four had stopped BF before two years. The fifth stated that her intention was to continue to BF for two years (ID-7).

### Reasons for stopping breast feeding

#### Working situation

Work was one of the main reasons for stopping EBF before the recommended six months and for not continuing with BF for two years. As one person explained “[work is the] biggest breastfeeding challenge . . . [it is] not convenient for mothers to combine BF and work.” (KI-elder). Some of the first-time mothers felt that they either could not take time of work to BF, or that their workplace was not an appropriate place for a young baby or for BF. One mother who was able to EBF for six months continued to BF until her baby was 12 months and then stopped despite having a supportive workplace where she could take her infant (IDI-1). The reason given for this was that the baby had stopped sucking milk because the baby had "front teeth above and below and can bite food. The baby just did not want to suck" (IDI-3). She had also been advised by the doctor that when to stop BF depended largely on the baby.

At the time of the survey, another of the mothers who was able to EBF for six months had not returned to her work in a factory but reported she intended to BF for the recommended two years. This first-time mother was able to be absent from her work until she stopped BF, a privilege not accorded to mothers who bottle-fed their babies (IDI-7). A third mother, who worked in a factory, said that her husband took care of the baby while she was at work, including giving expressed breast milk (IDI-5). Despite expressing breast milk multiple times each day however, she also gave water to her baby not realising that this meant she was not EBF.

### Insufficient milk

Another important reason given for stopping BF was insufficient breast milk. According to the health staff interviewed, this could be remedied by mothers eating more, mothers letting the baby suck repeatedly and offering both breasts to the baby instead of one. If these methods did not work, they said they advised milk formula alongside breast milk. While mothers agreed that breast milk is “best in the first period of birth”, there was a general consensus that as the baby grew, breast milk contained insufficient nutrients. As one mother explained, “after many months formula milk is necessary. It has more nutrients and is better than breast milk at this time.” (FDG-mother). Another mother said that when she was on the postpartum ‘hot bed’ of embers she felt she had insufficient milk and worried that her baby was thirsty so she began giving her baby water “with a spoon, many times a day”. Other mothers also reported giving under six-month old babies water to drink.

### “Compressed nipple”

Compressed nipple, which seems from the description to be inverted nipple, was reported as a common but treatable problem without recourse to a doctor. Traditional methods such as using two sticks to grab the nipple and pull it out, or massaging fire ash around the breast when wet were reported as common. The midwives noted however, that for some mothers, inverted nipple could lead to termination of BF.

### Baby is old enough to eat “any food”

Most first-time mothers believe that BF could be stopped when the baby was around 1 year of age. By this age the mothers felt that their babies would be able to “eat anything adults eat” (IDI-12, 14). A common sentiment is reflected in this quotation from one of the midwives *“*the baby can eat anything it wants around 1–2 years. The mother can decide to stop because of this.” (FDG2 -midwives).

### The baby’s decision

One mother in Sisattanak district explained that at about one year her baby “stopped sucking milk itself. It had front teeth above and below and could bite food. The baby just did not want to suck.” (IDI-1). However, another mother found that allowing her baby to decide resulted in a longer BF period than she had initially planned. “[I] wanted to stop at 7 months to go back to work but the baby would not drink breast milk from the bottle.” (IDI-3)

### Family experiences and influences of community elders and others

Two mothers said they were influenced by the BF experiences of their mothers and relatives and had BF for the same time as their own mothers. In both districts, key informants suggested that elders often advocate for the early introduction of complementary foods despite this being contradictory to health professional advice. One mother said that her mother had secretly given honey mixed with water to her baby because she was scared the baby was not receiving enough nutrients (IDI-2). Whilst another mother agreed that doctors’ advice to EBF for six months was good, she said she had followed the advice of her mother who had told her “when you give food to the baby [it] is happy, this means it is right.” (IDI-4). Some of the elders also felt it was acceptable to give water to babies under six months, as one person explained “if the baby needs water but mothers do not give it, the baby will lose water from the brain, little by little.” (KI-elder) She went on to say:

“When the baby cries, what should do to make the baby sleep? After the mother tries to give something, the baby stops crying. Additional food makes the baby full, it sleeps. Not enough from breast milk.” (KI-elder)

Two of the mothers also reported being influenced by the advice of friends or neighbours. One woman for example, on the advice of a neighbour, started giving formula food at five months. (IDI-6). Mothers also mentioned the concerns about BF including fear of shrinking breasts and feeling embarrassed about BF at social events as reasons for stopping BF.

### Influences of health staff

In Sisattanak district, the key informant from the Ministry of Health explained that sometimes, in the event of certain operations, accidents or diseases, health staff may advise mothers to stop BF. Focus group midwives in Hatxayfong district agreed that illness of the mother was the most important reason for weaning. However, they explained that this is not a common occurrence. They gave examples of tuberculosis, HIV and a hyperthyroid patient who had to stop BF when her baby was four months old because of the anti-hyperthyroid drug she was taking. Some mothers also reported being advised by health staff to give water to their babies before six months.

## Discussion

In this study the first-time mothers, the elders and the health service providers generally had a positive perception of BF and initiation of BF was mainly in accordance of WHO guidelines. The use of colostrum indicates a change from Lao traditional postpartum practices in which colostrum is discarded and the first breast feed delayed for 2–3 days [19]. A cross-sectional study in 41 villages on the outskirts of Vientiane capital city also found high use of colostrum [19]. We found universal ANC attendance in the group of first-time mothers included in the in-depth interviews which is also consistent with the above cross-sectional study [19]. Some caution is needed however in comparing our small qualitative sample with these larger quantitative studies. Elsewhere, ANC attendance has been found to be an important factor in early initiation of BF [20-22] and in the present study, the level of education of the first-time mothers and access to ANC is likely to explain the acceptance of colostrum and early initiation. Most of the mothers however, limited their diet following traditional postpartum food practices. Barennes and colleagues found a similar finding in the above mentioned Lao study [19].

Despite the overall positive perception of BF, less than half of all mothers EBF for the full six months or continued to BF for two years. Difficulties in achieving EBF during the first six months of life and early weaning have been observed in both developed and developing countries, even where there is a high prevalence of BF initiation [23]. In Lao PDR only 26.4% of Lao are exclusively BF between 0-5 months [24]. In our study as elsewhere, one of the main reasons for the early cessation of EBF and early weaning related to maternal employment and the practicalities of combining EBF and work [2,25-29].

In our study, 14 of the 16 mothers individually interviewed, had either stopped or intended to stop BF at less than 20 months. One reason for this was perceived insufficient milk or the perception that the baby was feeding poorly. This has been observed across many cultures [29-31]. An accepted sign that the baby is not receiving enough milk includes frequent crying. In the present study, increased infant crying was often reported as a sign of insufficient breast milk and has been observed elsewhere in Lao PDR [24]. Other accepted signs of insufficient milk include very long feedings, inadequate weight gain and sporadic bowel movements [32]. In this study, these signs were not mentioned by the health staff, the first-time mothers or the elders. It may be that participants mistakenly relied on infant satisfaction cues to determine the adequacy of milk supply. The treatment options mentioned by the midwives, such as letting the baby suck repeatedly and offering both breasts to the baby however, are consistent with medical literature [32]. They did not however, mention the use of medications, which may be due to either a lack of knowledge or a lack of supply.

Early introduction of complementary foods and particularly water as a result of the mother’s beliefs or advice from significant others was common and has been observed elsewhere [28,29,33,34]. Overall, EBF was poorly understood and many of the respondents did not perceive giving water as being non-EBF. Another reason given by a midwife for possible discrepancies between recommended duration of EBF and reported duration in the present study was that previously the Government of Lao PDR had recommended EBF for four months. This recommendation was changed in the mid-2000s, following the World Health Assembly adoption of EBF from birth up to 6 months [35]. Few of the first-time mothers in this study reported having postnatal education or counselling which has been identified as important in positively influencing the duration and exclusivity of BF [23,36]. A systematic review of the literature found a combination of lay support and education plus professional support helped promote EBF for six months [23]. A study in Vietnam found key differences in mothers who worked and EBF for the first six months differed in specific ways from the other mothers. The mothers who were able to EBF (*n* = 5), all felt they had enough milk, knew the appropriate time to introduce foods and liquids, and were mostly supported in their decisions by local health workers, family members or their workplace [28].

Some health staff mentioned certain medical conditions such as HIV, anti-hyperthyroid drugs and tuberculosis as reasons for not BF. Whilst midwives were correct in identifying HIV as a medically acceptable reason to stop BF, this is only recommended if suitable replacement feeding is available [37]. Breastfeeding however may be able to be continued if the mother has tuberculosis [37]. It would be useful to ensure all health staff are aware of the specific conditions in which BF is contraindicated.

As with most research, our study has some limitations. One is that all respondents were Lao Loum, the main ethnic group in Lao PDR. Second, the study is based on interview data from the mothers and recall bias cannot be excluded. To minimise this, the study deliberately included in the inclusion criteria for first-time mothers, that the upper limit of the baby’s age at the time of the study should be 18 months. A possible third limitation is that one of the researchers was a male which may have inhibited some of the women. Nevertheless, the male doctor is an experienced and trained doctor and qualitative researcher used to working with women and no discomfort was observed. Fourth, in each district respondents reported ANC attendance and delivery by trained health staff and the sample cannot be seen as representative of the majority of Lao first mothers. In 2009 for example only 20% of mothers in the country had a skilled attendant present at birth [14]. Further, while the universal attendance of ANC by respondents is consistent with the Barennes, Simmala et al. (2009) study in Vientiane, the average ANC attendance in the country was only 35% in 2009 [14], although this may be increasing with the growth in urbanisation. In addition, the women in the individual interviews and FGDs were relatively well educated. Nevertheless, as a qualitative study the aim was not representativeness, but rather to gain an in-depth understanding of EBF and BF practices and factors which influence these practices. Further, triangulation of the qualitative data was achieved by using different methods (interviews and FGDs) and sources, increasing the trustworthiness of the study [17,38]. In addition, field notes, interview transcriptions and documented data analysis provide a research trail [21,25]. While there are limitations in the transferability of the study results, the study does provide important insights into BF practices in areas which are undergoing socio-economic change as Lao PDR continues its transition to a free-market economy.

## Conclusion

Overall the respondents in our study were positive towards BF and were well-informed. While in the two districts included in this study, some traditional practices were being maintained, others such as discarding colostrum were giving way to more contemporary, evidence based practices. Our results however, need to be understood in the context of Vientiane Capital City, an area undergoing rapid socio-economic change, including increased access to education, ANC and SBAs. As elsewhere, our study suggests that even where there is early initiation of BF and positive attitudes towards BF, achieving optimal BF practices is difficult and requires a range of interventions. Given the well-documented benefits of BF, further effort needs to be directed towards increasing the understanding of the benefits of EBF of first-time mothers, lay people and health professionals. Information and education strategies also need to target some of specific gaps in knowledge identified in this study including understanding of what EBF entails and appropriate timing of the introduction of water and supplementary foods. Reducing the non-knowledge factors is also important. Of particular importance is working with employers and developing supportive employment policies, including looking at ways of supporting women who are self-employed or who work in the informal economy. In rapidly expanding economies such as Lao PDR, it is likely that the demand for female workforce participation will increase and mothers will have to make vital decisions about how to feed their infants while they are at work. Research is also needed to identify the optimal combination of interventions to promote good BF practices, including identifying factors which facilitate or impede breastfeeding and how inhibiting factors can be addressed.

## Competing interests

The authors declare that they have no competing interests.

## Authors’ contributions

JB from the Nossal Institute (Australia) assisted with the development of this project. HP contributed to design the research project, data collection, and writing the preliminary report. VS assisted in the study design, data collection and synthesising data. JD assisted with analysing and synthesising the data. All authors read and approved the final manuscript and contributed equally to this work.

## Pre-publication history

The pre-publication history for this paper can be accessed here:

http://www.biomedcentral.com/1471-2393/13/223/prepub
